# Negative histology in cervical specimens obtained with the "see and treat" method among women at a referral center in Rio de Janeiro, Brazil: a cross-sectional study

**DOI:** 10.1186/s12905-021-01552-6

**Published:** 2021-12-07

**Authors:** Renata Pereira Teodoro, Danielle Scherer, Maria José de Camargo, Ana Carolina Carioca da Costa, Cecília Vianna de Andrade, Fábio Russomano

**Affiliations:** 1 Woman’s Health Care Area, National Institute of Woman’s, Child’s and Adolescent’s Health, Oswaldo Cruz Foundation, Av. Rui Barbosa, 716, Flamengo, Rio de Janeiro, RJ CEP 22250-020 Brazil; 2 Clinical Research Unit, National Institute of Woman’s, Child’s and Adolescent’s Health, Oswaldo Cruz Foundation, Rio de Janeiro, Brazil; 3 Anatomopathology and Cytopatology Coordination, National Institute of Woman’s, Child’s and Adolescent’s Health, Oswaldo Cruz Foundation, Rio de Janeiro, Brazil

**Keywords:** Cervical intraepithelial neoplasia, Colposcopy, Overtreatment, Negative histology, Rio de Janeiro, Brazil

## Abstract

**Background:**

According to the Brazilian Guidelines on Cervical Cancer Screening, women with cytopathologic diagnosis of high-grade intraepithelial lesion, abnormal colposcopic findings, fully visible squamocolumnar junction and age 25 years or older should be treated at the first visit (“see and treat—S&T”). The main limitation to this approach is the risk of overtreatment, identified by histology without preinvasive lesion. The objectives of this study were to identify the overtreatment rate in women undergoing S&T in cervical cancer prevention at a referral center with extensive experience with the method and to detect possible factors associated with this rate.

**Methods:**

This was a cross-sectional study that analyzed records from a database with 616 women submitted to S&T from 1996 to 2017. Negative histology was defined as the following histopathologic results: human papillomavirus without cervical intraepithelial neoplasia (CIN), inflammatory, low-grade squamous intraepithelial lesion, and CIN 1.

**Results:**

Of the 616 women, there were 52 (8.44%, 95%CI 6.25–10.64%) with a histopathologic report without preinvasive cervical lesion. No statistical association was found between this outcome and age or a significant downward trend over time.

**Conclusion:**

The overtreatment rate in this study can be considered low and consistent with the acceptable rates reported in the literature, reinforcing the prevailing Brazilian guideline, in which the benefits of immediate treatment outweigh the risk of losses following biopsy.

## Background

In Brazil, there were an estimated 16,370 new cases of uterine cervical cancer per year in 2018 and 2019, with an estimated risk of 15.43 cases per 100 thousand women. Cervical neoplasms rank third among cancers in women. Worldwide, cervical cancer is the fourth most frequent tumor in women, with approximately 500,000 cases diagnosed per year, 70% of which in areas with low human development, where 9 out of all 10 deaths from cervical cancer occur [1].

The screening strategy for cervical cancer and precursor (or preinvasive) lesions in Brazil is periodic Papanicolaou or Pap smear test (cervical cytopathology), which has contributed to the reduction of cervical cancer incidence and mortality in recent decades, especially in developed countries. According to the World Health Organization (WHO), a coverage of at least 80% of the target population and adequate diagnosis and treatment of identified cases would allow reducing in the incidence of invasive cervical cancer by 60–90% [1].

According to the Brazilian Guidelines for Cervical Cancer Screening, women with high-grade squamous intraepithelial lesion (HSIL) and atypical squamous cells of undetermined significance in which it is not possible to rule out high-grade intraepithelial lesion (ASC-H) require immediate referral for colposcopy, due to the risk of invasive disease and possible need for treatment of a precursor lesion [2].

Immediate treatment of precursor lesions is recommended due to the risk of an undetected invasive lesion or to avoid progression to cancer [3]. This recommendation does not apply to situations in which the risks of treatment outweigh the benefits, as in pregnant or young women (under 25 years) [2].

The “see and treat” method (S&T) consists of an outpatient excisional treatment at the first visit. According to the Brazilian Guidelines, S&T is recommended in women aged at least 25 years-old, with a cervical cytopathology showing HSIL, in the presence of major abnormal colposcopic findings, that is, consistent with a preinvasive lesion, the disease is limited to the ectocervix or the squamocolumnar junction (SCJ) does not extend more than one centimeter inside the cervical canal, and in the absence of colposcopic characteristics suggestive of invasive or glandular disease. S&T is also acceptable in women in the same conditions but with ASC-H cytology. As an alternative to S&T, biopsy at the first visit is also considered acceptable, and in case of confirmation of high-grade cervical intraepithelial neoplasia (CIN 2 or CIN 3), the recommendation is to proceed to excisional treatment in a second visit [2].

S&T has operational advantages, reducing the time to treatment, ensuring fewer losses to follow-up and reducing the psychological and emotional impact associated with the diagnosis by allowing rapid resolution of the problem. The strategy is especially important for women that are unable to adhere to a follow-up plan such as watch-and-wait or treatment at a later date, after the biopsy result. The literature reports the method’s acceptability and feasibility when performed according to the appropriate criteria at center where the quality of cytology can be ensured and colposcopy is performed by experienced professionals, a situation in which the negative histology rate is similar to that of treatment in two stages with biopsy [4].

The main limitation to patient management with S&T is the risk of overtreatment, with a negative or low-grade histology i.e., the operative specimen having no preinvasive lesion. Overtreatment rates vary greatly in the literature. A meta-analysis of 13 studies found an overtreatment rate of 11.6% in cases with agreement between cytology and colposcopy, both suggestive of high-grade lesion, which is consistent with the negative histology rates found in women submitted to prior biopsy (11–35%). The overtreatment rate was higher when the test results were discordant [4]. This limitation is due to the variability in the performance of cytology and colposcopy, both of which are examiner-dependent [5].

Despite evidence in favor of S&T and the recommendation in the Brazilian Guidelines, its routine use is not widespread in Brazil, with negative impacts both for women and for the effectiveness of cervical cancer control programs, with possible losses of treatment opportunities and unnecessary expenditures.

The objectives of this study were to identify the overtreatment rate in women undergoing S&T in cervical cancer prevention at a referral center with extensive experience with the method and to detect possible factors associated with this rate.

## Methods

This was a cross-sectional study that included women submitted to S&T in a referral center in Rio de Janeiro, Brazil, from 1996 to 2017, referred from primary healthcare units in the same city and from other units in the state of Rio de Janeiro.

### Inclusion and exclusion criteria

Women at least 25 years-old, with HSIL or ASC-H cytology, transformation zone [TZ] types 1 or 2, and major abnormal findings on colposcopy, without prior biopsy were studied. Women with positive HIV serology (which has a higher prevalence of preinvasive lesions or cancer), pregnant women, or women with missing data were excluded.

### Procedures

During the study period, women that underwent cervical cytopathology at a public primary health unit in Rio de Janeiro and had an abnormal result (HSIL, ASC-H, HSIL cannot rule out microinvasive cancer, Abnormal Glandular Cells, Invasive Carcinoma, Adenocarcinoma in situ or after two results of Low-grade Squamous Intraepithelial Lesion or Atypical Squamous Cells of Undetermined Significance) were routinely referred for colposcopy and for other diagnostic and therapeutic procedures at a more complex center. At the first visit in the colposcopic clinic, women with cytopathology results of HSIL or ASC-H, aged 25 years or older and who were not pregnant received an informed consent form and were informed about the possibility of the treatment at the time of colposcopy in case they met the criteria stated in the Brazilian Guidelines. In these cases, the women were submitted to type 1 or 2 excision under local anesthesia and direct colposcopic view on the same day they arrived at the service or on a later date if they were menstruating or presenting a vaginal inflammatory process (after it’s treatment), or at their convenience.

Colposcopic findings were recorded by experienced colposcopists and classified according to the Colposcopic Terminology of the International Federation for Cervical Pathology and Colposcopy [6], and the histopathological diagnoses were made by experienced pathologists using the Richart classification [7].

### Data analysis and storage

Results were presented as mean, range and standard deviation (SD) for numerical variables. Categorical variables were expressed as counts, percentages and 95% confidence intervals (CI). Pearson’s chi-square test was used to analyze the association between cytopathology results and patient’s age up to 24 years, or up to 29 years. Binary logistic regression was used to estimate the odds ratio (OR) and to predict the probability of being a negative histology (which represents overtreatment) based on the year of treatment (predictor).

Data were stored in a local database. Missing data was recovered from hard-copy patient chart, covering a period from October 1996 to December 2017.

### Outcomes

Overtreatment was defined as procedures that resulted in histopathologic diagnosis of CIN 1, LSIL, HPV with CIN, or inflammatory, that is, without proven presence of preinvasive lesion diagnosed on referral cytopathology and suspected on colposcopy by the abnormal findings.

To verify a potential reduction in negative histology as more experienced the group turned to be, the researchers analyzed the distribution of negative diagnoses rates over the years.

## Results

From October 1996 to December 2017, the colposcopy center received 1,298 women, of whom 618 women were initially included. Two women were excluded, one because it proved impossible to confirm the approach that had been performed (since her patient chart had been misplaced), and the other because the patient had taken the specimen from the excisional procedure to have it processed elsewhere. Of the 616 remaining women, mean age was 31.55 years (range 15–54; SD 7.49). All of them were public health system users. Only one had a cytopathological diagnosis of ASC-H, and the others were HSIL.

Considering the remaining 616 women, 52 (8.44%, 95%CI 6.25–10.64%) had negative histopathology or CIN 1/HPV without CIN (Table 1).

**Table 1:** Distribution of histopathologic diagnoses in a sample of women submitted to S&T from 1996 to 2017, Rio de Janeiro, Brazil

Histopathologic diagnosis	N	%	95%CI^e¶^
AIS*	2	0.32	0–0.77
CIN^†^ 2	164	26.62	23.13–30.11
CIN 3	389	63.15	59.34–66.96
CIN 3 + AIS	5	0.81	0.10–1.52
Ca IA1^‡^	2	0.32	0–0.77
Subtotal with preinvasive or microinvasive (positive)	562	91.23	89.0–93.47
CIN I/HPV^§^ without CIN	45	7.31	5.25–9.36
Negative	7	1.14	0.30–1.97
Subtotal without preinvasive or microinvasive (negative)	52	8.44	6.25–10.64
Inconclusive/altered^||^	2.0	0.32	0–0.77
	616	100	

*Adenocarcinoma in situ

^†a^Cervical intraepithelial neoplasia

^‡^Microinvasive carcinoma (FIGO IA1)

^§^Cytopathic alterations related to human papillomavirus without intraepithelial neoplasia

^||^Inconclusive histopathologic diagnosis or compromised by thermal artifacts

^¶^95% confidence interval

Among the histopathology results defined as positive, 2 (0.32%) showed diagnosis of adenocarcinoma in situ (AIS) and 2 (0.32%) were squamous cell carcinoma, stage IA1. The others were CIN 2–3 or CIN 3 associated with AIS. The total number of preinvasive or microinvasive (positive) lesions was 562 cases (91.23%, 95%CI 89.0–93.47%). Only 2 cases (0.32%) showed an inconclusive histopathological diagnosis or diagnosis compromised by thermal artifact (Table 1).

**Fig. 1:**
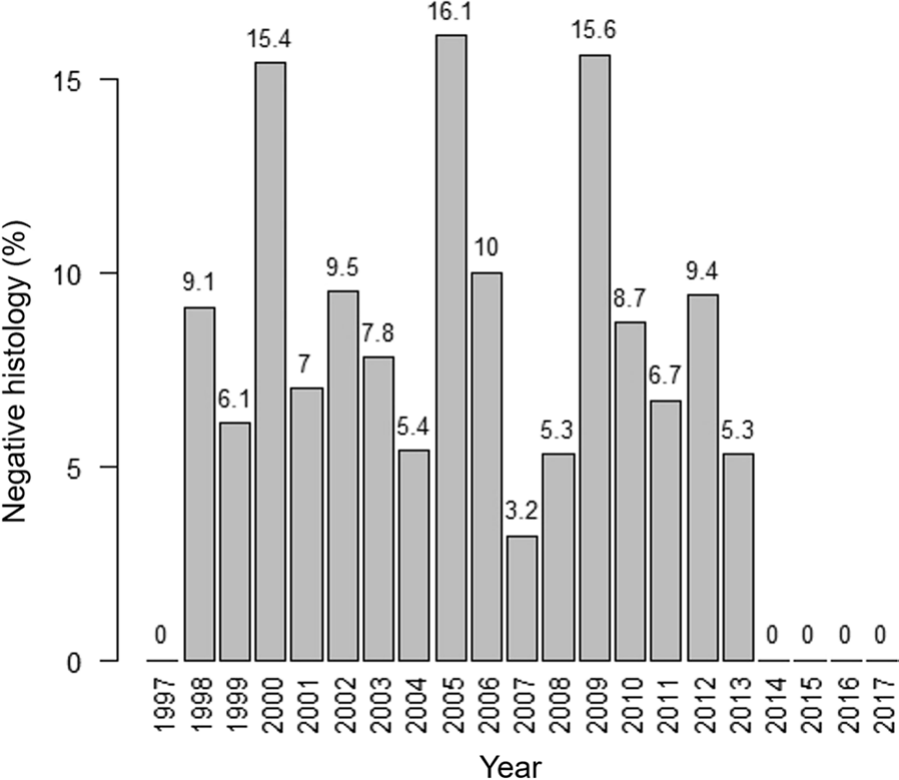
Percentages of negative histology according to the year of treatment—1997 to 2017, Rio de Janeiro, Brazil

To check the trend of negative histologies over the years, we analyzed the percentages of negative diagnoses according to the year of treatment, excluding the year of 1996 (which had only one of negative histology case included) (Fig. 1).

**Fig. 2:**
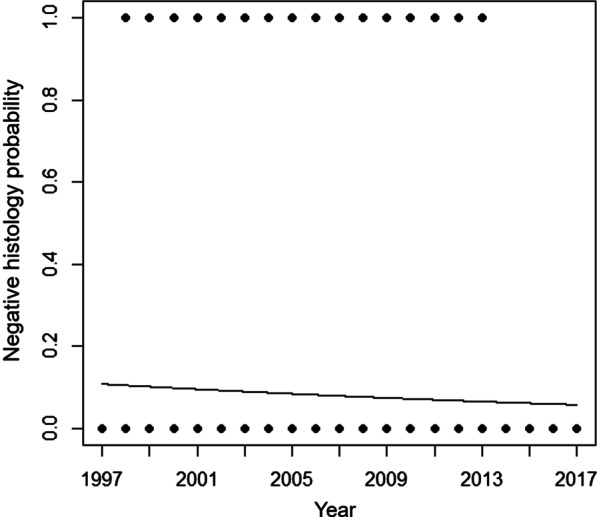
Probability of negative histology as a function of year in which the procedure was performed—1997 to 2017, Rio de Janeiro, Brazil

To verify whether there was a reduction in the probability of overtreatment over the years, a binary logistic regression was adjusted with year of treatment as the predictor. The results showed a slight downward trend in the probability of negative histology over the years, although no statistical significance was found (crude OR = 0.97, 95%CI 0.91–1.02). The year of 1996 were also excluded from this analysis (Fig. 2).

## Discussion

Findings from this study are consistent with what is expected in the Brazilian Guidelines for Cervical Cancer Screening, which chose the S&T method as the best option for women that meet the procedure’s eligibility criteria.

The overtreatment rate when performed in two stages, with prior biopsy, ranges from 11 to 35%, a variability attributed to the limitation of intra- and inter-observer agreement on the biopsy and histopathology results from the excised specimen [4]. These values may also be due to excisional biopsies. The colposcopy center where the current study was performed is considered a referral center in the city of Rio de Janeiro, with highly experienced professionals, and is a teaching and research institute, thus corroborating the centres’ quality.

In a meta-analysis that aimed to estimate overtreatment in women undergoing S&T, in 13 studies that included 3403 women with high-grade cytology and colposcopy, the overtreatment rate was 11.6% [5]. In this study, overtreatment was defined as CIN 1 or less, as in our study, in which we found an even lower rate (8.7%). Seven studies from this same meta-analysis and including 374 women with high-grade cytology and low-grade colposcopy showed that the overtreatment rate was even higher (29.3%), as expected in this situation. Meanwhile, studies including 506 women and showing low-grade cytology and high-grade colposcopy found an overtreatment rate of 46.4%. In the group of studies with 328 women with low-grade cytology and colposcopy, overtreatment was 72.9% [5].

We emphasize that CIN 2 is considered here as a preinvasive lesion since the present Brazilian Guidelines recommends its treatment because of its unknown prognosis. Although women with this histopathologic result are not as prevalent as the other with CIN 3 someone would be concerned about the use of S&T in younger women. That´s why this approach is not recommended for women younger than 25 years old in Brazil. In fact, since recent papers had put into light the high probability of spontaneous regressions of CIN 2 the previous biopsy approach had become the first choice in women younger than 30 years old, with less marked alterations or with lesions limited to one quadrant of the ectocervix.

Importantly, the effectiveness and appropriate application of the S&T method requires adequate criteria in its indication, and agreement between the cytology and colposcopy findings is essential. In cases of disagreement between cytology and colposcopy, we believe it is prudent to perform a biopsy, with treatment reserved for confirmed cases of high-grade intraepithelial lesion, especially in young and fertile women, to avoid unnecessary treatment that can have a negative impact on their reproductive future, besides other unwanted effects.

Despite the observed risk of overtreatment, S&T offers other benefits for women, such as prompt resolution of the problem, diminishing both the waiting time and the number of return visits to the outpatient clinic, as well as fewer expenses for both the patient and the health service, which is thus able to increase the uptake of new women. A previous study in the same service, from 1998 to 2004, found the same negative histology rate (8.7–2.0% negative results and 6.7% LSIL, CIN 1, and HPV without CIN), in a sample of 298 women submitted to S&T [8].

If one considers the CIN 1 histology rate acceptable, given the observed cytologic and colposcopic alterations in these cases, in which the excisional procedure met the diagnostic objective, the negative histology rate would be 3.3% (95%CI 1.9–4.7), reinforcing the prevailing recommendation, in which the benefits of immediate treatment outweigh the risks of losses following biopsy.

We attempted to correlate negative histology with younger patient age and the reduction of negative histology percentages over time, as the result of the team’s accumulated experience. However, the results were not statistically significant, and we could thus not identify groups at risk of negative histology or the team’s greater experience as capable of avoiding this outcome (despite the absence of negative histology results in the last 4 years of the study).

We further emphasize that a reduction in the probability of negative histology resulting from the team’s accumulated experience, although not statistically significant, is a plausible hypothesis and deserves attention, since the percentages of negative histology rates were lower on average starting in 2007 compared to the earlier years (4.9% in recent years, versus 8.6% before 2007).

## Conclusions

The overtreatment rate in this study can be considered low and consistent with the acceptable rates reported in the literature. Being adherent to recommended criteria for applying S&T, i.e., when the woman has a cervical cytology showing HSIL and major alterations at colposcopy with SCJ completely visible (TZ types I or II), can reduce unnecessary treatment of lesions that would otherwise present spontaneous regression, or even in the absence of lesions.

## Data Availability

The datasets used and/or analyzed during the current study are available from the corresponding author on reasonable request.

## Abbreviations

ASC-H: Atypical squamous cells of undetermined significance in which it is not possible to rule out high-grade intraepithelial lesion
CIN: Cervical Intraepithelial Neoplasia
HSIL: High-grade squamous intraepithelial lesion
LSIL: Low-grade squamous intraepithelial lesion
S&T: See and treat method
SCJ: Squamocolumnar junction
